# Analysis of therapy monitoring in the International Congenital Adrenal Hyperplasia Registry

**DOI:** 10.1111/cen.14796

**Published:** 2022-07-11

**Authors:** Neil Lawrence, Irina Bacila, Jeremy Dawson, Jillian Bryce, Salma R. Ali, Erica L. T. van den Akker, Tânia A. S. S. Bachega, Federico Baronio, Niels H. Birkebæk, Walter Bonfig, Hedi C. van der Grinten, Eduardo C. Costa, Liat de Vries, Heba Elsedfy, Ayla Güven, Sabine Hannema, Violeta Iotova, Hetty J. van der Kamp, María Clemente, Corina R. Lichiardopol, Tatjana Milenkovic, Uta Neumann, Ana Nordenström, Şukran Poyrazoğlu, Ursina Probst‐Scheidegger, Luisa De Sanctis, Rieko Tadokoro‐Cuccaro, Ajay Thankamony, Ana Vieites, Zehra Yavaş, Syed Faisal Ahmed, Nils Krone

**Affiliations:** ^1^ Department of Oncology and Metabolism University of Sheffield Sheffield UK; ^2^ Sheffield Children's Hospital NHS Foundation Trust Sheffield UK; ^3^ Institute of Work Psychology, Management School University of Sheffield Sheffield UK; ^4^ School of Health and Related Research, University of Sheffield Sheffield UK; ^5^ Office for Rare Conditions Royal Hospital for Children & Queen Elizabeth University Hospital Glasgow UK; ^6^ Office for Rare Conditions Royal Hospital for Children & Queen Elizabeth University Hospital Glasgow UK; ^7^ Developmental Endocrinology Research Group University of Glasgow Glasgow UK; ^8^ Department of Pediatric Endocrinology, Sophia Children's Hospital Erasmus Medical Centre Rotterdam the Netherlands; ^9^ Hormones and Molecular Genetics Laboratory LIM 42, Department of Internal Medicine University of Sao Paulo Sao Paulo Brazil; ^10^ Department of Medical and Surgical Sciences, Pediatric Unit, Endo‐ERN Center for Rare Endocrine Diseases S. Orsola‐Malpighi University Hospital Bologna Italy; ^11^ Department of Pediatrics Aarhus University Hospital Aarhus Denmark; ^12^ Department of Pediatrics Technical University Munich Munich Germany; ^13^ Department of Pediatrics Klinikum Wels‐Grieskirchen Wels Austria; ^14^ Department of Pediatric Endocrinology Radboud University Medical Centre Nijmegen the Netherlands; ^15^ Amalia Children's Hospital Radboud University Medical Centre Nijmegen the Netherlands; ^16^ Pediatric Surgery Service Hospital de Clínicas de Porto Alegre Porto Alegre Brazil; ^17^ Institute for Diabetes and Endocrinology Schneider's Children Medical Center of Israel Petah‐Tikvah Israel; ^18^ Pediatrics Department Ain Shams University Cairo Egypt; ^19^ Baskent University Istanbul Hospital Pediatric Endocrinology Istanbul Turkey; ^20^ Department of Paediatric Endocrinology, Erasmus MC, Sophia Children's Hospital University Medical Center Rotterdam Rotterdam the Netherlands; ^21^ Department of Paediatrics Leiden University Medical Centre Leiden the Netherlands; ^22^ Department of Paediatrics Medical University of Varna Varna Bulgaria; ^23^ Pediatric Endocrinology Wilhelmina Children's Hospital University Medical Centre Utrecht Utrecht the Netherlands; ^24^ Paediatric Endocrinology, Hospital Universitario Vall d'Hebron CIBER de Enfermedades Raras (CIBERER) ISCIII Barcelona Spain; ^25^ Department of Endocrinology University of Medicine and Pharmacy Craiova Craiova Romania; ^26^ Department of Endocrinology Institute for Mother and Child Healthcare of Serbia “Dr Vukan Čupić” Belgrade Serbia; ^27^ Institute for Experimental Pediatric Endocrinology and Center for Chronically Sick Children, Charite‐Universitätsmedizin Berlin Germany; ^28^ Department of Women's and Children's Health Karolinska Institutet Stockholm Sweden; ^29^ Department of Paediatric Endocrinology, Astrid Lindgren Children Hospital Karolinska University Hospital Stockholm Sweden; ^30^ Istanbul Faculty of Medicine, Paediatric Endocrinology Unit Istanbul University Istanbul Turkey; ^31^ Pediatric Department Kantonsspital Winterthur Winterthur Switzerland; ^32^ Paediatric Endocrinology Regina Margherita Children's Hospital Torino Italy; ^33^ Department of Public Sciences and Pediatrics University of Torino Torino Italy; ^34^ Department of Pediatrics University of Cambridge, Cambridge, United Kingdom Biomedical Campus Cambridge UK; ^35^ Centro de Investigaciones Endocrinológicas (CEDIE‐CONICET), Hospital de Niños Ricardo Gutiérrez Buenos Aires Argentina; ^36^ Pediatric Endocrinology and Diabetes Marmara University Istanbul Turkey; ^37^ Department of Medicine III University Hospital Carl Gustav Carus, Technische Universität Dresden Dresden Germany

**Keywords:** biomarkers, congenital adrenal hyperplasia, hydrocortisone, linear mixed‐effects models

## Abstract

**Objective:**

Congenital adrenal hyperplasia (CAH) requires exogenous steroid replacement. Treatment is commonly monitored by measuring 17‐OH progesterone (17OHP) and androstenedione (D4).

**Design:**

Retrospective cohort study using real‐world data to evaluate 17OHP and D4 in relation to hydrocortisone (HC) dose in CAH patients treated in 14 countries.

**Patients:**

Pseudonymized data from children with 21‐hydroxylase deficiency (21OHD) recorded in the International CAH Registry.

**Measurements:**

Assessments between January 2000 and October 2020 in patients prescribed HC were reviewed to summarise biomarkers 17OHP and D4 and HC dose. Longitudinal assessment of measures was carried out using linear mixed‐effects models (LMEM).

**Results:**

Cohort of 345 patients, 52.2% female, median age 4.3 years (interquartile range: 3.1–9.2) were taking a median 11.3 mg/m^2^/day (8.6–14.4) of HC. Median 17OHP was 35.7 nmol/l (3.0–104.0). Median D4 under 12 years was 0 nmol/L (0–2.0) and above 12 years was 10.5 nmol/L (3.9–21.0). There were significant differences in biomarker values between centres (*p* < 0.05). Correlation between D4 and 17OHP was good in multiple regression with age (*p* < 0.001, *R* ^2^ = 0.29).

In longitudinal assessment, 17OHP levels did not change with age, whereas D4 levels increased with age (*p* < 0.001, *R* ^2^ = 0.08). Neither biomarker varied directly with dose or weight (*p* > 0.05). Multivariate LMEM showed HC dose decreasing by 1.0 mg/m^2^/day for every 1 point increase in weight standard deviation score.

**Discussion:**

Registry data show large variability in 17OHP and D4 between centres. 17OHP correlates with D4 well when accounting for age. Prescribed HC dose per body surface area decreased with weight gain.

## 1 INTRODUCTION

Congenital adrenal hyperplasia (CAH) is an autosomal recessive condition leading to glucocorticoid deficiency, androgen excess, variable degrees of mineralocorticoid deficiency, salt wasting and a risk of life‐threatening adrenal crisis. Poorly controlled CAH causes abnormal growth resulting in reduced adult height, reduced quality of life, increased comorbidities and shorter life expectancy.^1^, ^2^ Significant variation in treatment strategies has been noted in the United Kingdom and internationally, including using different formulations and dosing regimens.^3^ An international consensus statement in 2002^4^ was followed by a 2010 Endocrine Society guideline,^5^ updated in 2018, that improved guidance for clinicians,^6^ but there remain points of contention. The optimal balance of glucocorticoid, mineralocorticoid replacement, and need for salt replacement in infants is debated. It is acknowledged that treatment should be individualised, but precisely how to use the results from biochemical markers in the context of biometric measurements in children is unknown.^2^, ^7^

The recommended daily dose range of hydrocortisone (HC) is 10–15 mg/m^2^/day, with a recent review advocating doses up to 18 mg/m^2^/day.^7^ However, others suggest doses over 17 mg/m^2^/day should only be used with care during puberty as adult height has been shown to correlate negatively with glucocorticoid dose.^1^, ^2^, ^8^ Maintaining 17‐OH progesterone (17OHP) concentrations in the upper end of the normal range is suggested,^6^ with alternative targets including 17OHP of 10–20,^9^ 12–36^10^ or 3–36 nmol/l across all ages and sexes.^2^ Interpreting 17OHP and Androstenedione (D4) is challenging due to interindividual variability of their concentration profile in relation to glucocorticoid replacement, and variable practice in measurement in relation to timing of medication administration.^11^, ^12^ While alternative serum steroids^13^ and urinary steroids^14^ have been advocated for monitoring CAH, 17OHP and D4 are likely to be most frequently used in the medium term.

We analysed real‐world data from the International Congenital Adrenal Hyperplasia Registry (I‐CAH) (www.i-cah.org) to compare reported measurements of serum hormones in relation to prescribed doses of HC. We designed a longitudinal analysis, with repeated measures from patients managed in centres throughout different countries of the world, to gain insight into variations within patients as they age, between patients and to quantify the differences in results between different centres.

## 2 MATERIALS AND METHODS

### 2.1 Study design, setting and participants

This retrospective multi‐centre cohort study, including 21 centres (14 countries), analysed information on patients from the I‐CAH registry. The I‐CAH Registry is an international database of pseudonymised information on patients with CAH and is approved by the National Research Ethics Service in the United Kingdom as a research database of information that is collected as part of routine clinical care (19/WS/0131). The data within this registry are deposited by clinicians following informed consent from patients or guardians. Participants were under 19 years of age with a diagnosis of 21‐hydroxylase deficiency (21OHD) treated with oral HC as glucocorticoid replacement. All clinic visits that were recorded between January 2000 and October 2020 were analysed in this study. Data fields included in analysis are listed in Supporting Information: Table S1. Study design is limited by no overall quality assurance between the centres for laboratory assays, and variation in timing of sample collection and techniques of laboratory analysis and auxological assessments. This limitation is mitigated in part by advanced statistical analysis and separation of multilevel models into appropriate fixed and random effects.

### 2.2 Data analysis

Serum 17OHP and D4 of patients within different centres was summarised alongside their height, weight, and most recent dose of HC, and serum biomarkers compared between centres. Recommended range thresholds for 17OHP were 12–36 nmol/L as recommended before morning medication by Merke et al.,^10^ although it should be noted that there is no international consensus on a precise target range for 17OHP in CAH and the timings of measurement around morning dose within this cohort varied (alternative 10–20 nmol/L analysis in Supporting Information: Table S2).

The same variables measured in patients over time were analysed using linear mixed‐effects modelling (LMEM) to obtain insight into the within‐patients and between‐centres variability of HC dose and biomarkers. LMEMs are multilevel regression equations that allow stratification of different groups of data. Fixed effects are variables assumed to have a consistent effect across the whole cohort. Random effects are used to group aspects of the model that are interrelated, and thus have different coefficients that apply to each of the separate groups of data.^15^ The model intercepts varied based on the random effects in our models of patient at level 1 and treatment centre at level 2. The fixed effects of each model are described within the results. This is not an appropriate technique to model nonlinear data, and thus height was not modelled.

Total daily dose of HC was expressed per BSA, calculated using the Mosteller formula.^16^ Weight was converted to age and sex‐adjusted standard deviation scores (SDS) by using The Growth Analyser software version 4.1.5 against World Health Organisation (WHO) international multicentre growth reference study normative data. Participating centres were contacted to confirm which units were used when entering biomarker data into the registry, and all biomarker measurements were converted to nmol/l. To restrict analysis to patients established on glucocorticoid replacement, primary clinic visits within 3 months of diagnosis were excluded.

Statistical analysis was performed using *R: A language and environment for statistical computing* (R Core Team, R Foundation for Statistical Computing, https://www.R-project.org/). The biomarkers 17OHP and D4 exhibit positive skew, thus reported as median, with interquartile range (IQR). Paired comparisons were carried out using the Mann–Whitney test, and group comparisons with the Kruskal–Wallis test. Bayesian multiple change point analysis was used to select subcategories of ages for cross‐sectional analysis. *R* ^2^ values for LMEMs represent the proportion of variance explained by both fixed and random effects.

## 3 RESULTS

### 3.1 Cross‐sectional analysis

#### 3.1.1 Cohort characteristics

Analysing each patient's most recent biomarkers was necessary to avoid patients with more data points having a disproportionate influence on summary statistics. This produced a cohort of 345 patients, 52.2% female, aged median 4.3 years (IQR: 3.1 to 9.2). Patients had a median weight SDS of 0.3 (IQR: −1.1 to 1.7) and were taking a median HC dose of 11.3 mg/m^2^/day (IQR: 8.6 to 14.4). Biomarkers reported in the registry below the lower limit of detection of a centre's assay are rounded to zero. Median 17OHP was 35.7 nmol/l (IQR: 3.0 to 104), 15.9% within a target of 12–36 nmol/L and 50.0% above this range, and median D4 was 0 nmol/L (IQR  0 to 3.5). Median 17OHP was inside the tighter control range of 10–20 nmol/l in just 0.6% of patients (Supporting Information: Table S2). Bayesian change‐point analysis confirmed no suitable age categorisation for 17OHP, but a change point for D4 of 12 years, thus summary statistics were produced for those under and over 12 years (Supporting Information: Figure 1). There was significant difference between those under 12 years (17OHP median 29.0 nmol/L [IQR: 3.0 to 93.0] and D4 median 0 nmol/L [IQR: 0 to 2]) versus those 12 years and over (17OHP median 60.50 nmol/L [IQR: 29.0 to 151.0] and D4 median 10.5 nmol/L [IQR: 3.9 to 21.0]) [Table 1] (*p* < 0.001)]. However, differences in weight and dose are noted and thus interpreting causality from comparisons between these cross‐sectional cohorts is inappropriate, and instead warrants advanced statistical modelling.

**Table 1:** Summary statistics of individual patients' most recent recorded clinic visit

	Total	Male patients	Female Patients	Age 0–12	Age 12–18
Number of patients	345	165	180	283	62
Age (years) Median (IQR)	4.3 (3.1 to 9.2)	4.2 (3.0 to 8.0)	4.5 (3.1 to 10.8)	3.7 (2.7 to 6.1)	14.5 (13.3 to 15.8)
Weight SDS median (IQR)	0.26 (−1.07 to 1.70)	0.31 (−1.01 to 1.93)	0.18 (−1.15 to 1.60)	−0.20 (−1.22 to 0.98)	2.59 (1.99 to 3.34)
Dose of hydrocortisone per BSA per day (mg/m^2^/day)	11.3 (8.6 to 14.4)	11.6 (8.4 to 14.4)	11.1 (8.7 to 14.1)	10.9 (8.4 to 13.8)	13.3 (10.6 to 15.5)
Number with 17OHP reading	334	160	174	277	57
17OHP (nmol/l) Median (IQR)	35.7 (3.0 to 103.7)	33.0 (6.0 to 93.2)	40.0 (3.0 to 120.3)	29.0 (3.0 to 93.0)	60.5 (29.0 to 151.0)
Percentage with 17OHP < 12 nmol/l	34.1	33.1	35.1	37.9	15.8
Percentage with 17OHP between 12 and 36 nmol/l	15.9	17.5	14.4	16.6	12.3
Percentage with 17OHP > 36 nmol/l	50.0	49.4	50.6	45.5	71.9
Number with D4 reading	298	149	149	243	55
D4 (nmol/l) (IQR)	0 (0 to 3.5)	0 (0 to 3.5)	0 (0 to 5.0)	0 (0 to 2.0)	10.5 (3.9 to 21.0)

Abbreviations: 17OHP, 17‐OH progesterone; BSA, body surface area; D4, androstenedione; IQR, interquartile range; SDS, standard deviation score.

#### 3.1.2 Comparison between sexes, centres and time periods

**Figure 1:**
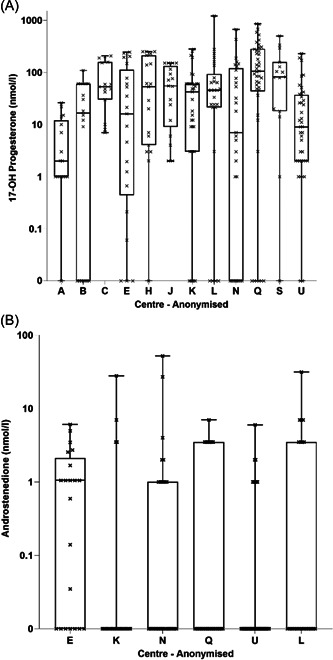
Comparison of each patient's most recent serum biomarkers between different centres. Absolute values plotted on logarithmic scale. Only centres with 10 or more patients with each biomarker are displayed. Horizontal lines correspond to median. Kruskal–Wallis comparison shows significant difference between centres (*p* < 0.05). (A) 17‐OH Progesterone, (B) androstenedione measured in patients under 12 years. Only 2 centres had 10 or more patients over 12 with D4 levels available for comparison, and Kruskal–Wallis comparison revealed no difference (*p* = 0.76).

There was no significant difference in dose, weight SDS, BMI SDS, or biomarker readings between males and females (*p* > 0.05) (Table 1). Comparing the pooled results from all centres in 5 year periods (2001–2005, 2006–2010, 2011–2015 and 2016–2020), there was no difference in median 17OHP or D4 concentrations in the last 20 years (*p* > 0.05). Comparing centres with over 10 readings available, there were differences in median 17OHP, ranging from a low median within centre of (2.0 nmol/l [IQR: 1.0 to 10.0] up to a high median within centre of 104.4 nmol/l [IQR: 46.1 to 273.9] [*p* < 0.001]). Patients under 12 had variable D4, ranging from a low median within centre of 0 (0 to 0) to a high median within centre of 1.0 (IQR: 0 to 1.7) (*p* = 0.013) (Figure 1), but no significant difference between D4 readings in those over 12 years, where only 2 centres had sufficient readings for comparison (*p* = 0.76). The variance of the measurements of 17OHP, but not D4, was significantly different between centres (Levene's test, *p* < 0.001).

#### 3.1.3 Correlation between biomarkers

There was good correlation between 17OHP and D4, strongest when controlling for patient age (Table 2). Correlation was stronger in patients under 12 years (*p* < 0.001, *R* ^2^ = 0.24), with readings in patients over 12 years not quite statistically significant (*p* = 0.07, *R* ^2^ = 0.07). Correlation between these markers was similar in both sexes. Multiple regression of D4 against 17OHP covaried with age revealed consistent correlation between 17OHP and D4 (D4 = 0.608 x Age + 0.025 x 17OHP − 1.56, *p* < 0.001, *R* ^2^ = 0.29) showing age should be considered when interpreting these markers together.

**Table 2:** Bivariate correlation between the biomarkers D4 and 17‐OH Progesterone

Dependent variable	D4 (nmol/l) in patients under 12 years old	D4 (nmol/l) in male patients under 12 years old	D4 (nmol/l) in female patients under 12 years old	D4 (nmol/l) in patients 12 years and older	D4 (nmol/l) in male patients 12 years and older	D4 (nmol/l) in female patients 12 years and older
Number with paired readings	235	123	112	49	21	28
Intercept	0.65	0.36	1.00	11.54	12.87	9.99
*p*‐value of intercept	0.138	0.539	0.136	<0.001	0.010	<0.001
17OHP coefficient	0.024	0.027	0.022	0.018	0.025	0.018
*p*‐value of 17OHP	<0.001	<0.001	<0.001	0.068	0.327	0.065
*R* ^2^	0.237	0.286	0.189	0.0692	0.050	0.125

Abbreviations: 17OHP, 17‐OH progesterone; D4, androstenedione.

### 3.2 Longitudinal analysis

#### 3.2.1 Cohort available for modelling

Longitudinal assessment within patients was carried out to assess how biomarkers, weight and dose changed within patients with age. A total of 308 patients (50% female) from 21 centres on HC replacement with 2707 visits between 2000 and 2020 were available for longitudinal modelling, 1813 visits with biomarker data available and 1642 visits with dose data available. Median age at visit was 3.2 years (IQR: 1.7 to 6.1), maximum age at visit was 18.7 years, with the median number of visits available per patient being 7 (IQR: 5 to 10.5).

#### 3.2.2 Univariate LMEM analysis

**Figure 2:**
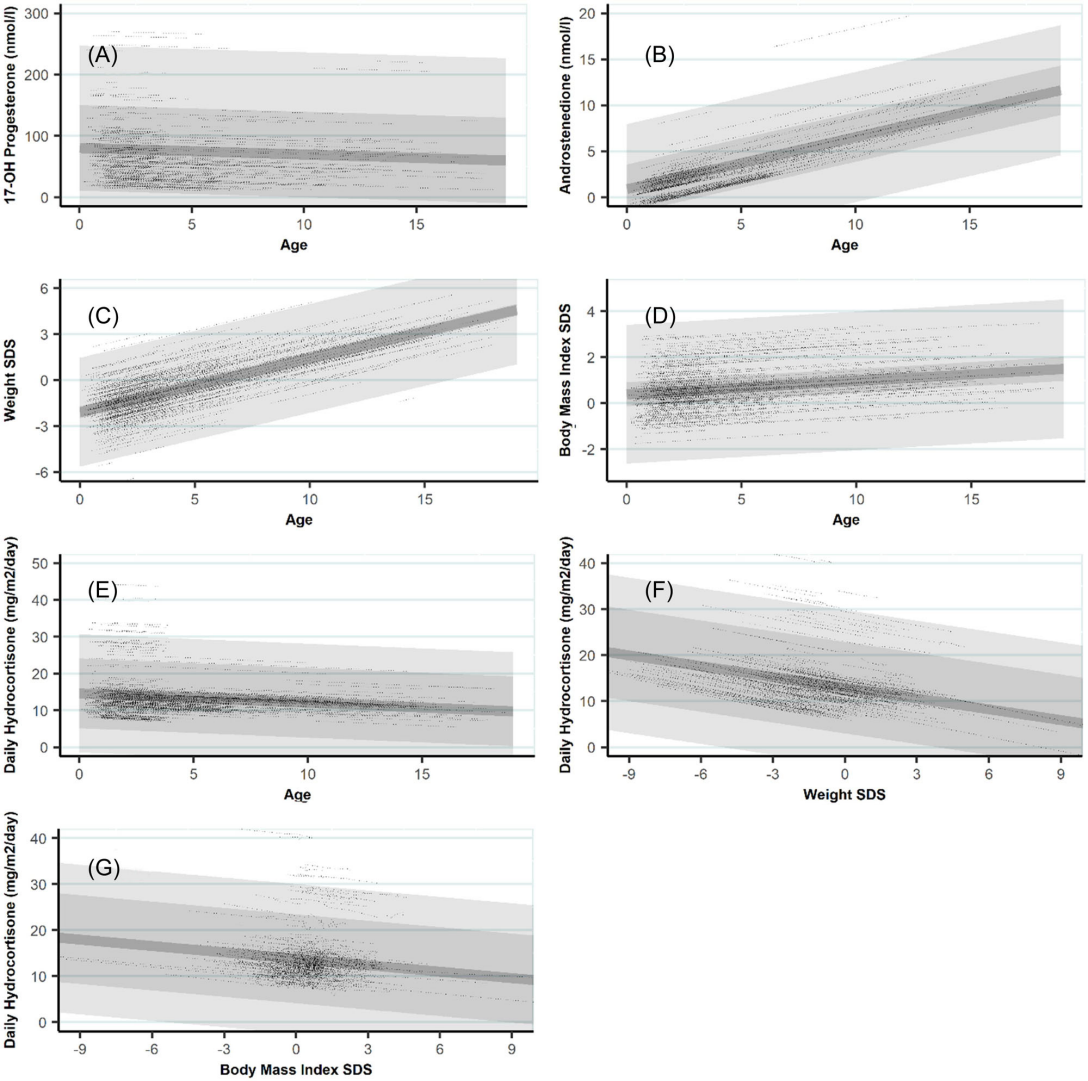
Linear mixed effect models with random intercepts. Random effects stratify data into patient treated at level 1, and treatment centre at level 2. Dark line shows overall model fit. Central dark shading shows 95% confidence interval attributable to treatment centre, outer lighter shading shows remaining proportion attributable to individual patients. Dotted lines show individual model fit to each patient. (A) 17OHP on age. (B) Androstenedione on age. (C) Weight Standard Deviation Score (SDS) on age. (D) Body Mass Index (BMI) on age. (E) Total Daily Hydrocortisone (HC) on age. (F) Covariation of Weight SDS and Total Daily HC modelled with age. (G) Covariation of BMI SDS and Total Daily HC modelled with age.

We found an increase in D4 with age, an increase in weight and BMI standard deviation score (SDS) with age and a decrease in dose relative to BSA with age (Table 3). Serum 17OHP showed a trend towards a decrease with patient age, although not statistically significant (*p* = 0.33, conditional *R* ^2^ = 0.20). Serum D4 increased by 0.56 nmol/l per year of age (*p* < 0.001, *R* ^2^ = 0.08). Weight SDS increased on average by 0.35 per year of age (*p* < 0.001, *R* ^2^ = 0.44) and BMI SDS by 0.06 per year of age (*p* = 0.002, *R* ^2^ = 0.56), although the rate of increase was greater in those under 5 years. Dose relative to BSA decreased by 0.26 mg/m^2^/day per year of age within patients (*p* < 0.001, *R* ^2^ = 0.38) (Figure 2).

**Table 3:** Linear mixed‐effects model parameters with univariate fixed effects and random intercepts

Dependent variable	17OHP (nmol/l)	D4 (nmol/l)	Weight SDS	BMI SDS	Hydrocortisone per BSA (mg/m^2^/day)
Intercept	80.51	0.86	−2.09	0.39	14.62
*p*‐value of intercept	<0.001	0.256	<0.001	0.002	<0.001
Age coefficient	−1.07	0.56	0.35	0.06	−0.26
*p*‐value of age coefficient	0.332	<0.001	<0.001	<0.001	<0.001
SD of patient random effect	49.52	2.25	1.61	1.27	3.36
Patient ICC	0.64	0.70	0.98	0.96	0.33
SD of centre random effect	36.87	1.48	0.24	0.27	4.83
Centre ICC	0.36	0.30	0.02	0.04	0.67
Conditional *R* ^2^ a	0.20	0.08	0.32	0.56	0.38
Corresponding figure	2A	2B	2C	2D	2E

*Note*: Random effects stratify data into patient treated at level 1, and treatment centre at level 2.

Abbreviations: 17OHP, 17‐OH progesterone; BSA, body surface area; D4, androstenedione; ICC, intraclass correlation coefficient; SD, standard deviation; SDS, standard deviation score.

^a^ Conditional *R* ^2^ = proportion of the variance explained by both fixed and random effects.

Studying the variance within these models attributed to the centre suggests that differences in practice were most apparent in total dose, with consistent differences also in biomarkers. The intraclass correlation coefficient (ICC) shows the proportion of variance within the model that is attributable to individual patients versus treatment centre. Most of the change in weight and BMI SDS with age is dependent upon the individual patient (ICC = 0.98 and 0.96, respectively), indicating the weight gain seen with age is consistent across all centres. With dose, patient ICC is 0.33, confirming that different patients require different doses, but twice as much of the variance is attributable to the centre (ICC = 0.67), indicating consistently different dosing practices. The centre effect accounted for approximately one‐third of the variance of biomarkers between patients (17OHP: ICC 0.36, D4: ICC 0.30).

#### 3.2.3 Multivariate LMEM analysis

As univariate LMEMs showed increasing D4 concentrations within patients with age, and total daily dose of HC decreasing with age, we added covariates to investigate confounding (Table 4). Weight SDS was added to each univariate model, and biomarkers then added to create multivariate LMEMs.

**Table 4:** Linear mixed‐effects model parameters with multivariate fixed effects random intercepts

Dependent variable	17OHP	D4	Hydrocortisone dose per BSA (mg/m^2^/day)	Hydrocortisone dose per BSA (mg/m^2^/day)	Hydrocortisone dose per BSA (mg/m^2^/day)	Hydrocortisone dose per BSA (mg/m^2^/day)
Intercept	79.02	0.81	12.39	12.87	13.03	14.82
*p*‐value of intercept	<0.001	0.332	<0.001	<0.001	<0.001	<0.001
Age coefficient	−0.82	0.57	0.085	0.13	−0.0024	−0.23
*p*‐value of age coefficient	0.546	<0.001	0.186	0.187	0.984	<0.001
Weight SDS coefficient	−0.770	−0.022	−1.024	−1.015	−0.974	Not in model
*p*‐value of weight SDS coefficient	0.726	0.908	<0.001	<0.001	<0.001	Not in model
BMI SDS coefficient	Not in model	Not in model	Not in model	Not in model	Not in model	−*0.47*
*p*‐value of BMI SDS coefficient	Not in model	Not in model	Not in model	Not in model	Not in model	*<0.001*
17OHP coefficient	Not in model	Not in model	Not in model	−0.0001	Not in model	Not in model
*p*‐value of 17OHP coefficient	Not in model	Not in model	Not in model	0.937	Not in model	Not in model
D4 coefficient	Not in model	Not in model	Not in model	Not in model	0.0037	Not in model
*p*‐value of D4 coefficient	Not in model	Not in model	Not in model	Not in model	0.867	Not in model
SD of patient random effect	50.02	2.28	3.571	3.233	3.578	3.365
Patient ICC	0.65	0.70	0.32	0.24	0.28	0.32
SD of centre random effect	36.87	1.48	5.197	5.785	5.690	4.927
Centre ICC	0.35	0.30	0.68	0.76	0.72	0.68
Conditional *R* ^2^ a	0.20	0.08	0.44	0.37	0.35	0.39
Corresponding figure	‐	‐	2F	‐	‐	2G

*Note*: Random effects stratify data into patient treated at level 1, and treatment centre at level 2.

Abbreviations: 17OHP, 17‐OH progesterone; BSA, body surface area; D4, androstenedione; ICC, intraclass correlation coefficient; SD, standard deviation; SDS, standard deviation score.

^a^ Conditional *R* ^2^ = proportion of the variance explained by both fixed and random effects

Weight was significantly associated with dose in multivariate analysis and showed that the relationship between dose decreasing with age was due to the confounding effect of increasing weight SDS with age. When weight SDS was added as a covariate to the model of HC dose relative to BSA against age, HC dose per BSA decreases by 1.02 mg/m^2^/day for every 1 point increase in their weight SDS. Similarly, HC dose per BSA decreased by 0.47 mg/m^2^/day for every 1 point increase in BMI SDS (Figure 2).

Importantly, neither 17OHP nor D4 when added as covariates to the strongest model were statistically significant (Table 4, *p* > 0.05), meaning we could not show any biochemical evidence using individual hormone measurements that this altered the level of disease control. Patient height is nonlinear and therefore not appropriate to add to this random intercept LMEM.

## 4 DISCUSSION

We reviewed real‐world data from the I‐CAH Registry in patients under 19 years of age taking HC for 21OHD to evaluate the markers 17OHP and D4 in relation to HC dose. There was large variability, with 17OHP commonly above target range and D4 increasing with age, with significant variability between treatment centres. Repeated measures analysis with LMEMs showed that patients are treated with lower HC doses per BSA as their weight and BMI SDS increases with age, a novel finding not previously reported. Addition of biomarkers to this longitudinal model reduced the model fit to the data, meaning that this relationship was not accompanied by deterioration in control detectable through isolated measurements of serum biomarkers.

The biomarker 17OHP varies with age and sex in healthy children,^17^ with guidance that values in CAH may be above the normal range in patients with adequate control.^4^ Recent guidelines and reviews suggest that normalising 17OHP inappropriately is an indication of overtreatment in CAH, without specifying a precise target.^6^, ^7^ Alternative target ranges are based on expert opinion and include 3–36 or 12–36 nmol/l for 17OHP, with advice to normalise D4 into the sex and age‐specific range.^10^ The median 17OHP we report here is at the upper end of these target ranges with large interindividual variability. Painful phlebotomy can influence 17OHP,^18^ thus variability will in part be due to heterogeneity in sampling techniques and timing of blood tests in relation to HC administration between different centres. These data highlight the difficulty in interpreting isolated measurements and therefore the importance of holistic patient assessment.

Both 17OHP and D4 have a short half‐life, vary throughout the day and relative to treatment administration, 17OHP having greater variability than D4.^19^, ^20^ Some centres perform multiple measurements of serum steroids to accurately predict their 24 h profile.^12^, ^19^, ^21^ Alternatives such as 21‐deoxycortisol have been investigated for the diagnosis of CAH, but not routinely used for monitoring treatment.^13^ Urinary steroid profiles can contribute to disease monitoring, although thresholds need validating in larger patient populations.^14^ Serum 11‐hydroxy‐testosterone and 11‐Ketotesterone have been shown to discriminate well between poor and good control in CAH. However, it has been suggested they perform better in adults than children.^22^ In our extraction of data for modelling, 66% of clinic assessments had either 17OHP or D4 measured, indicating the high prevalence of their use. Developing the evidence base of how best to interpret these hormones when taken as point measurements is important, and while interpretation is difficult, they will likely remain the most frequently used biomarkers of disease control for the foreseeable future.

While 17OHP varies with age in healthy children,^17^, ^23^ our cross‐sectional analysis shows higher levels in the cohort over 12 years. However, 17OHP does not vary with age in our more sophisticated longitudinal analysis, indicating the importance of the LMEM and telling us that clinicians are aiming for similar values of 17OHP throughout childhood. D4 increases with age in our cohort of patients, as it does in healthy children due to the gonadal production of androstenedione after puberty,^17^, ^24^ and agrees with data from controlled trials.^25^ We demonstrate concordance between D4 and 17OHP, that is strongest when accounting for the increase of D4 with age. The proportion of variation of this correlation explained by our model (*R* ^2^ = 0.3) is less than that in other highly controlled single centre studies (*R* ^2^ = 0.7).^26^ This is likely due to the larger age range of our patients and larger variance exhibited in the biomarker results themselves, which will in turn be a combination of greater variability in compliance with treatment and possible data entry errors. The correlation we see indicates there is likely clinical benefit from measuring both markers in patients to assess disease control, but that age must be considered when interpreting the results. Further research using appropriate multivariate and longitudinal analysis to establish optimal age‐ and sex‐specific D4 targets in patients with CAH would be beneficial to improve clinical utility.

Biochemical markers of control are variable between centres, but do not correlate with dose of HC. One‐third of the variance of biomarkers within patients was attributable to the centre managing treatment, rather than individual patients requiring different doses. When the biomarkers measured are incorporated into the model describing the variation of dose with weight SDS, they are not significant, showing that differences in doses between centres are not directly related to the serum biomarkers, even when accounting for age and weight SDS. This may be due to the different populations and genetic differences,^2^ or because of differences in height or pubertal status that are not accounted for in our models, but highlights the limitation of interpreting isolated measurements of these hormones drawn at different times. The use of different assays and variable timing of clinic appointments and blood tests, as well as different target ranges for biomarkers may be the cause of some centre variation. However, as the centre contributed an even larger two‐thirds of the variance within the dosing model, we can conclude that there is significant variation in practice between centres that is resulting in varying levels of serum biomarkers and doses of replacement glucocorticoids.

Recommended replacement dose of HC since an international consensus statement in 2002 has been 10–15 mg/m^2^ per day, although others since have recommended higher ranges, such as 12–18 mg/m^2^ per day,^7^ and a meta‐analysis has shown children with CAH commonly prescribed 15–20 mg/m^2^ per day of HC.^27^ Doses exceeding 15–20 mg/m^2^ per day are associated with growth suppressing effects.^7^ Our median dose of 11.3 mg/m^2^ per day is within the recommended range, although the wide range of shows some still being treated with significantly higher doses of HC than would be deemed necessary.

Our longitudinal analysis shows the confounding effect that a general trend towards the weight SDS of children with CAH to increase as they age has on the dose they are receiving. The multivariate analysis shows that the alteration in dose is related to this change in weight, resulting in a 1.0 mg/m^2^/day decrease in dose per 1 point increase in weight SDS, a similar trend persisting with BMI. This is evidence of the complex interrelationship between growth in children with CAH, disease control, and required replacement dose of glucocorticoid. Puberty is also likely to have a significant effect on this relationship, when they are known changes in cortisol pharmacokinetics.^28^ Coexistent polycystic ovary syndrome in adolescent females may lead to higher values of D4.^29^ Puberty was not included in our analysis because of the large proportion of this data that are at an age before pubertal development.

Due to the short half‐life of HC and the high variability in clearance,^11^ as well as the variability seen in endogenous 21‐hydroxylase activity in patients with CAH, it is accepted that different patients are likely to need different doses of replacement to achieve the same level of disease control.^2^ The fact that adding either of the biomarkers 17OHP or D4 into our model describing the variation of dose with age and weight was markedly statistically insignificant suggests that different patients will require different doses of glucocorticoid replacement to maintain appropriate disease control. Current dose per BSA should therefore not impact upon the clinical assessment of disease control unless it is to consider the possibility of poor compliance.

The statistical techniques and large sample size strengthen this study and show that interpreting summary statistics in different populations of patients with CAH can lead to potentially inaccurate conclusions. Simply interpreting the average dose in younger patients as lower than older patients (Table 1) fails to acknowledge the possibility of confounding differences in covariates, most markedly the difference in their weight and BMI SDS. Assessment of repeated measures in a multilevel model inherently controls for confounding factors between patients as we are assessing how our different metrics are changing within the same patients over time, and show in this case that dose is decreasing as their weight and BMI SDS increases (Figure 2).

Values reported in this study are those that clinicians are faced with in routine practice and rely upon to make clinical decisions, which gives us valuable insight into these metrics outside of the confines of controlled settings. Limitations associated with this data from different centres include different laboratories using different assays, and blood drawn at different times. Differences in demographics and prevalence of different genotypes in different countries will contribute to differences in the metrics studied that will not exclusively be due to differences in clinical practice. The small number of large outlying values may indicate noncompliance with treatment or data entry errors.

In conclusion, children with increasing weight and BMI SDS are being prescribed less glucocorticoid dose per BSA. Assessment of biochemical markers within this relationship has not shown clear detriment to their disease control, although this warrants further investigation in relation to a more holistic assessment of control. Dose should be regularly reviewed taking into consideration their growth, pubertal development, biomarkers, side effects of treatment and compliance. Standard biomarker measurement practices are needed to evaluate biochemical evidence of disease control. Collection of real‐world data within the established I‐CAH platform is to be encouraged and will allow us to gain further insights into patients as they progress through puberty, helping to improve patient care and reducing unwarranted variation in practice.

## CONFLICT OF INTEREST

The authors declare no conflicts of interest.

## Supporting information

Supporting information.

## Data Availability

The datasets generated or analysed during the current study are not available publicly but available to access through a data sharing agreement available at home.i-cah.org.
