# Supplementary material for: Analysis of therapy monitoring in the International Congenital Adrenal Hyperplasia Registry
Source: Clin Endocrinol (Oxf). 2022 Jul 11;97(5):551–61. doi: 10.1111/cen.14796 (PMC9796837; doi:10.1111/cen.14796)

**Supplementary Table 1**

Data fields extracted for analysis in this study

| **Register ID** |
| --- |
| **Centre Name** |
| **Country** |
| **Sex at birth** |
| **Date of Diagnosis** |
| **Date (CAH Longitudinal Data)** |
| **Weight (kg) (CAH Longitudinal Data)** |
| **Height (cm) (CAH Longitudinal Data)** |
| **Age at visit** |
| **Has treatment changed since last visit** |
| **Glucocorticoids – current medication type** |
| **Glucocorticoids – current medication dose** |
| **Androstenedione value** |
| **17-OH Progesterone value** |

**Supplementary Table 2**

Proportion of 17OHP readings related to the alternative clinical threshold to assess disease control of 10-20nmol/l

|  | **Total** | **Male patients** | **Female Patients** | **Age 0-12** | **Age 12-18** |
| --- | --- | --- | --- | --- | --- |
| Number with 17OHP Reading | 334 | 160 | 174 | 277 | 57 |
| 17OHP (nmol/l)  Median (IQR) | 35.7  (3.0 to 103.7) | 33.0  (6.0 to 93.2) | 40.0  (3.0 to 120.3) | 29.0  (3.0 to 93.0) | 60.5  (29.0 to 151.0) |
| Percentage with 17OHP < 10nmol/l | 32.9 | 32.5 | 33.3 | 36.8 | 14.0 |
| Percentage with 17OHP between 10-20nmol/l | 0.6 | 0.6 | 0.6 | 0.4 | 1.8 |
| Percentage with 17OHP > 20nmol/l | 66.5 | 66.9 | 66.1 | 62.8 | 84.2 |

**Supplementary Figure 1**

Bayesian Multiple change point analysis of Androstenedione with age on most recent patient readings. This analysis was used to select appropriate sub populations to report summary statistics in table 1


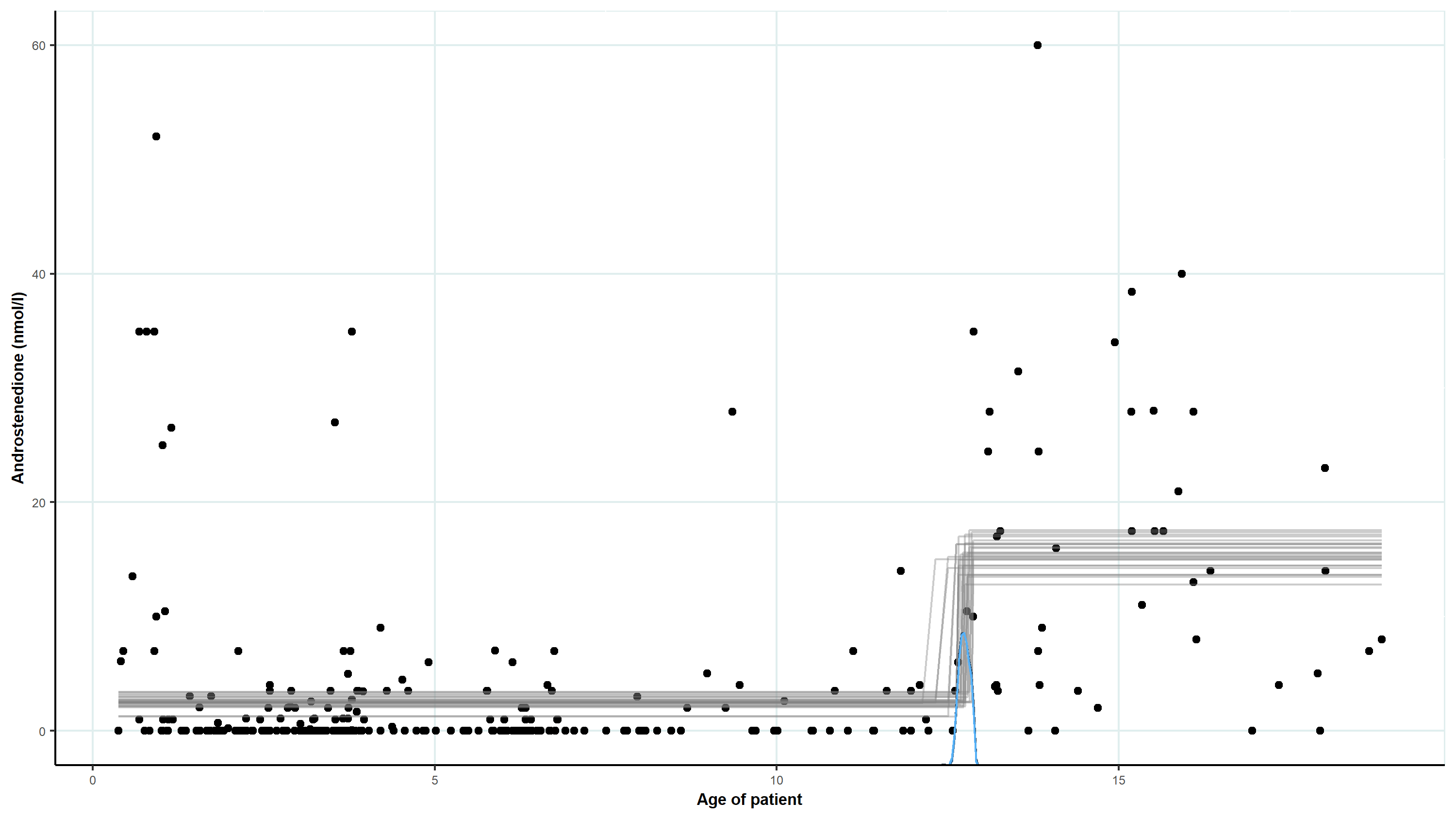

Supplement: Supplementary file 1 — Supporting information. [file CEN-97-551-s001.docx]
